# Embedding locally tailored antimicrobial prophylaxis into standardized order sets for urological procedures reduces antimicrobial use

**DOI:** 10.1093/jacamr/dlag202

**Published:** 2026-09-03

**Authors:** Darunee Chotiprasitsakul, Punrapee Srichompoo, Kun Sirisopana, Kumthorn Malathum

**Affiliations:** Division of Infectious Diseases, Department of Medicine, Faculty of Medicine Ramathibodi Hospital, Mahidol University, 270 Rama VI Road, Thung Phaya Thai, Ratchathewi, Bangkok 10400, Thailand; Division of Infectious Diseases, Department of Medicine, Faculty of Medicine Ramathibodi Hospital, Mahidol University, 270 Rama VI Road, Thung Phaya Thai, Ratchathewi, Bangkok 10400, Thailand; Division of Urology, Department of Surgery, Faculty of Medicine Ramathibodi Hospital, Mahidol University, Bangkok, Thailand; Division of Infectious Diseases, Department of Medicine, Faculty of Medicine Ramathibodi Hospital, Mahidol University, 270 Rama VI Road, Thung Phaya Thai, Ratchathewi, Bangkok 10400, Thailand

## Abstract

**Introduction:**

Inappropriate selection and dosing of antimicrobial prophylaxis (AP) for urological procedures remain common. We therefore evaluated the impact of a locally adapted AP guideline, embedded within standardized pre-procedure order sets and supported by antimicrobial stewardship interventions, on antimicrobial utilization in a high-resistance setting.

**Methods:**

A 1-year study of the three phases—pre-intervention, intervention and after intervention—was performed at a tertiary care hospital in Thailand. The intervention included development of local AP guidelines based on the institutional antibiogram, integration into one-page order sets, multifaceted education and feedback. Antimicrobial utilization was assessed using days of therapy (DOT) per 1000 patient-days. Interrupted time series (ITS) analysis was used to evaluate changes in antimicrobial use over time. Postoperative urinary tract infections (UTIs) and surgical site infections (SSIs) within 30 days were monitored.

**Results:**

A total of 657 urological procedures were included. Median total DOT per admission declined from 10 days (IQR 8–12) to 2 days (IQR 1–4) (*P* < 0.001). The proportion of patients receiving single-dose AP increased from 1.29% pre-intervention to 33.66% post-intervention (*P* < 0.001). ITS analysis demonstrated a significant immediate reduction in weekly total DOT per 1000 patient-days following intervention implementation (level change −3156.8; 95% CI −4604.7 to −1708.9), with no significant change in slope. The incidence of SSI and UTI remained below 1% across all periods.

**Conclusions:**

Implementation of locally tailored AP guidelines integrated into standardized order sets, supported by stewardship interventions, was associated with a substantial and sustained reduction in antimicrobial exposure for urological procedures. This approach is relevant to high-resistance, resource-limited settings.

## Introduction

Antimicrobial prophylaxis (AP) is routinely administered for urological procedures to reduce the risk of postoperative infections. However, inappropriate use of prophylactic antimicrobials, including suboptimal agent selection and unnecessarily prolonged duration, remains common, with particularly high rates of inappropriate AP reported in low- and middle-income countries (LMICs).^1–4^ These practices contribute to avoidable antimicrobial exposure and may accelerate antimicrobial resistance without improving patient outcomes.^5^

International guidelines may have limited applicability in the Thai community due to differences in local antimicrobial susceptibility patterns of Gram-negative uropathogens.^6,7^ At our institution, nearly one-third of community-onset Gram-negative bloodstream infections are not susceptible to third-generation cephalosporins.^8^ In high-resistance settings, broadening prophylactic coverage to target prevalent resistant pathogens may help reduce infectious complications. A retrospective propensity score–matched study at our institution found that carbapenem prophylaxis was associated with a significantly lower incidence of early extended-spectrum cephalosporin-resistant UTI within 14 days compared with cefuroxime.^9^ However, agent selection must be carefully weighed against the risk of accelerating resistance through indiscriminate broad-spectrum antimicrobial use.

Antimicrobial stewardship programmes (ASPs) support this balance by implementing procedure-specific guidelines and standardized order sets that improve prophylaxis appropriateness, streamline workflow and minimize adverse effects.^10^

The primary objective of this study was to evaluate the impact of a multicomponent ASP intervention on antimicrobial utilization for urological procedures, as measured by changes in agent selection and duration. Secondary objectives included describing postoperative SSI and UTI rates within 30 days.

## Methods

### Clinical setting and study population

This study was conducted at Ramathibodi Hospital, a 1300-bed tertiary care hospital in Bangkok, Thailand. The study population included all consecutive patients admitted for urological procedures between 1 January 2024 and 31 December 2024. Exclusion criteria were pre-procedural infection, kidney transplantation, procedure postponement and participation in another study. The study was divided into three consecutive 4-month phases: a baseline observation period (Period 1: 1 January to 30 April 2024), an intervention period (Period 2: 1 May to 31 August 2024) and a follow-up period (Period 3: 1 September to 31 December 2024).

### Definitions

SSI and UTI were defined according to the Centers for Disease Control and Prevention/National Healthcare Safety Network surveillance definitions.^11,12^ Antimicrobial utilization was quantified using days of therapy (DOT) per 1000 patient-days.

### Data collection

The following data were collected from medical records: demographics, pre-existing medical conditions, diagnosis, type of urological procedure and the occurrence of SSI and UTI within 30 days after the procedure, before and after the intervention. Information on antimicrobial prescribing, including the antimicrobial agent, prescribing frequency, duration of therapy, number of doses administered and admission and discharge dates, was extracted from the hospital business intelligence system. Antimicrobial administration was reviewed from before the urological procedure until patient discharge.

### Intervention design

Period 1 baseline data were retrospectively collected. A locally adapted AP guideline was developed collaboratively by representatives from the Division of Infectious Diseases and the Division of Urology, incorporating international guideline recommendations,^5–7,13,14^ local epidemiology and the institutional antibiogram (Figure S1, available as Supplementary data). Routine pre-procedural urine cultures were not incorporated into prophylaxis decision-making, as obtaining cultures within 1–2 weeks prior to procedure was not feasible within the existing clinical workflow. Previous urine culture results, when available and recognized, could inform the selection of AP at the discretion of the treating urologist.

**Figure 1.**
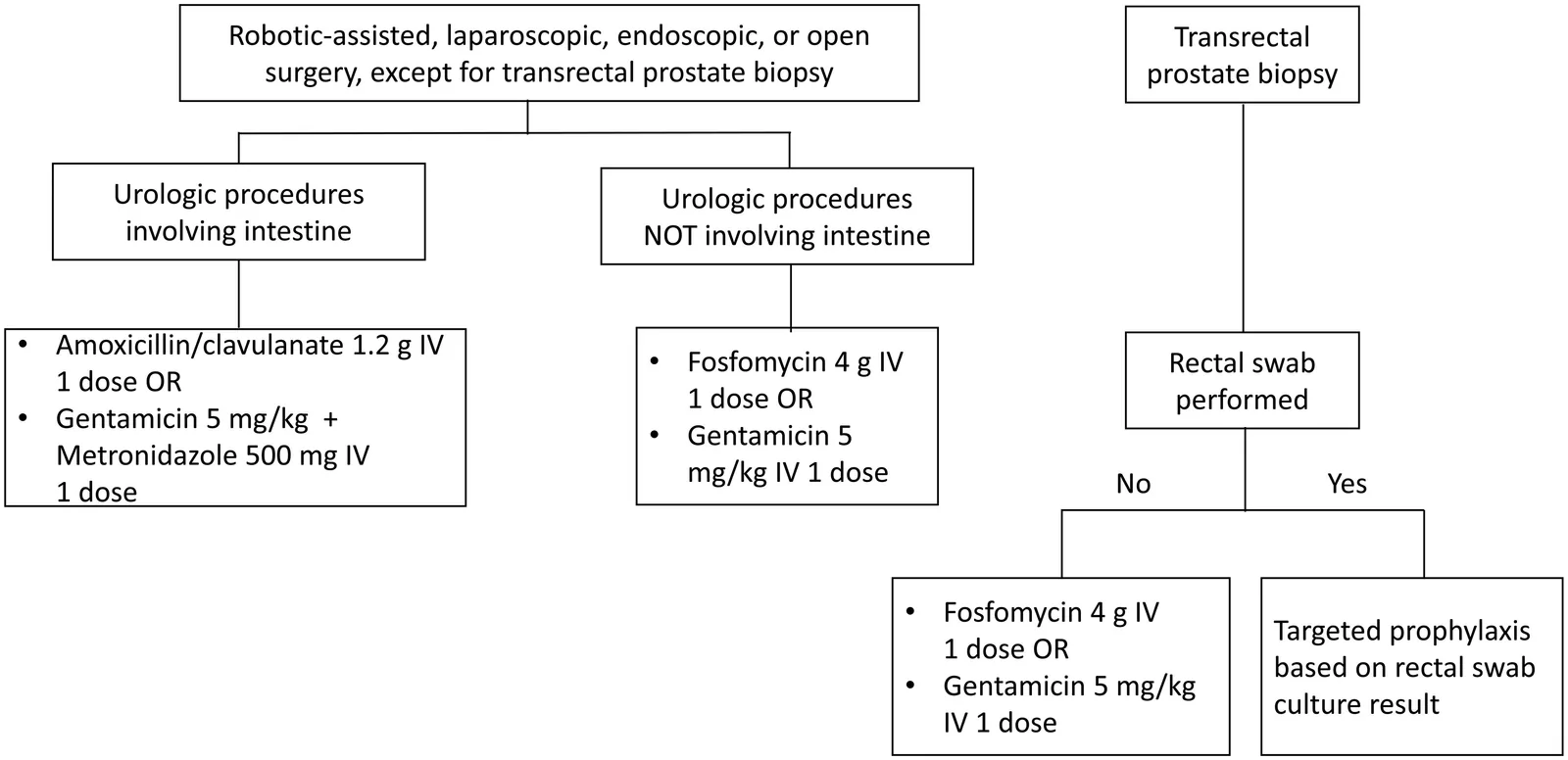
Flow of antimicrobial prophylaxis based on the type of urological procedure and the institutional antibiogram.

Recommended agents were selected based on expected pathogen spectrum by procedure type, local susceptibility data and their relatively limited use in treatment settings to preserve antimicrobial activity (Figure 1). For procedures not involving the gastrointestinal tract, Gram-negative uropathogens are the predominant organisms. The local antibiogram demonstrated >80% susceptibility to amoxicillin/clavulanate, fosfomycin and gentamicin. Fosfomycin has activity against extended-spectrum cephalosporin-resistant Gram-negative organisms and Gram-positive organisms, including *Enterococcus* and *Staphylococcus*.^15^ Clinical studies have demonstrated the efficacy of both oral and intravenous fosfomycin for urological AP.^16,17^ Given its very high activity against locally prevalent uropathogens and relatively limited use for the treatment of extended-spectrum cephalosporin-resistant infections compared with carbapenems and piperacillin/tazobactam, fosfomycin was selected as the preferred agent in the local guideline. Gentamicin was retained as an alternative agent to provide Gram-negative coverage. For procedures involving the gastrointestinal tract, amoxicillin/clavulanate or a combination of gentamicin plus metronidazole was recommended to ensure adequate anaerobic coverage. Targeted prophylaxis for transrectal prostate biopsy based on rectal swab culture was included as an additional option.

The guideline was integrated into one-page pre-procedure order sets (Figure S2), organized into five sections: laboratory investigations, pre-operative instructions, pre-anaesthetic orders, surgical preparation and AP regimens (specifying agent, route, dose, timing and duration). The order sets were evaluated for practicality in a meeting with the urology staff and residents, during which feedback and suggestions were provided. The final order sets were reviewed and approved by the Institutional Medical Records Committee prior to implementation.

Period 2 Implementation began with two interactive educational sessions on urological infections for urology physicians. These sessions also emphasized the guideline rationale, order sets and prior non-adherence patterns. Outpatient and inpatient nurses in the Division of Urology were reminded during routine staff meetings to ensure placement of the order form in the admission charts. A study team member conducted weekly compliance reviews in the urology ward. A secure messaging group was established between the urology residents and infectious diseases physicians to facilitate reminders, feedback and support interdepartmental consultations.

Period 3 involved data collection only. No active interventions were conducted, and no feedback was provided during this phase.

### Statistical analysis

Categorical variables were compared between the three periods using the chi-square test or Fisher’s exact test as appropriate. Continuous variables were expressed as mean ± standard deviation or median (interquartile range, IQR) as appropriate, with comparisons by one-way ANOVA or Kruskal–Wallis H test. *Post hoc* pairwise comparisons against Period 1 used Bonferroni correction when global *P* < 0.05.

An interrupted time series (ITS) analysis using segmented regression was performed to evaluate temporal changes in antimicrobial utilization across 52 weekly observations.^18^ The model estimated (i) the pre-intervention baseline trend, (ii) the immediate level change at the time of intervention implementation and (iii) the post-intervention trend change. A transitional window during initial order sets rollout (weeks 20–23) was excluded from trend estimates. Autoregressive error structures were incorporated, and residual autocorrelation was assessed using the Durbin–Watson statistic. All tests were two-tailed, and *P* < 0.05 was considered statistically significant. Analyses were performed using Stata v18.0 (Stata Corp., College Station, TX, USA).

### Ethical approval

The study was approved by the Institutional Review Board of the Faculty of Medicine Ramathibodi Hospital, Mahidol University (approval number: MURA2024/228). As this work was conducted as part of quality improvement activities within the standard of care, the requirement for written informed consent was waived. All prescribing and clinical management decisions remained under the authority of the primary attending physicians.

## Results

### Patient characteristics

There were 709 patients admitted for urological procedures (Table 1). Of these, 52 patients were excluded due to participation in another research study or the absence of a urological procedure. Therefore, 657 patients (232 in Period 1, 223 in Period 2 and 202 in Period 3) were included in the analysis. Patient characteristics are summarized in Table 1. The median age of patients in Period 2 was significantly older than in Period 1 (70 years, IQR 61–76 versus 67 years, IQR 56–73; *P* = 0.01). Most patients were male (71%). Pre-existing comorbidities were comparable across periods. The distribution of most urological procedures was similar across the three periods. The most common procedure was transurethral resection of bladder tumour (13.36%, 16.59% and 14.85% in Periods 1–3, respectively). However, the proportion of transurethral prostatectomy was significantly different (10.34%, 13.00% and 18.81%; *P* = 0.04).

**Table 1.** Clinical characteristics of patients undergoing urological procedures by study period

Variables	Period 1 (n = 232)	Period 2 (n = 223)	Period 3 (n = 202)	*P*
Age, years (IQR)	67 (56–73)	70 (61–76)^a^	68 (58–74)	0.01
Gender, n (%)				0.71
Male	165 (71.12)	160 (71.75)	138 (68.32)	
Female	67 (28.88)	63 (28.25)	64 (31.68)	
Length of stay, days (IQR)	3 (2–5)	3 (2–5)	3 (2–5)	0.12
Pre-existing medical conditions, n (%)				
Diabetes mellitus	49 (21.12)	68 (30.49)	51 (25.25)	0.07
Hypertension	142 (61.21)	146 (65.47)	118 (58.42)	0.32
Chronic kidney disease	31 (13.36)	37 (16.59)	35 (17.33)	0.47
Cardiovascular disease	32 (13.79)	35 (15.7)	32 (15.84)	0.80
Liver disease	6 (2.59)	8 (3.59)	6 (2.97)	0.82
Cerebrovascular disease	15 (6.47)	23 (10.31)	15 (7.43)	0.30
Immunocompromised state	15 (6.47)	16 (7.17)	8 (3.96)	0.34
Malignancy	32 (13.79)	30 (13.45)	22 (10.89)	0.62
Principal diagnosis, n (%)				
Calculus of kidney and ureter	34 (14.66)	35 (15.70)	36 (17.82)	0.66
Malignant neoplasm of prostate	34 (14.66)	35 (15.70)	22 (10.89)	0.33
Malignant neoplasm of bladder	33 (14.22)	31 (13.9)	31 (15.35)	0.91
Hyperplasia of prostate	30 (12.93)	35 (15.7)	46 (22.77)^a^	0.02
Obstructive and reflux uropathy	16 (6.90)	17 (7.62)	10 (4.95)	0.52
Malignant neoplasm of kidney, except renal pelvis	13 (5.6)	10 (4.48)	4 (1.98)	0.16
Disorders of bladder	12 (5.17)	15 (6.73)	7 (3.47)	0.33
Other primary diagnosis	60 (25.86)	45 (20.18)	46 (22.98)	0.35
Procedure, n (%)				
Transurethral resection of bladder tumour	31 (13.36)	37 (16.59)	30 (14.85)	0.62
Transurethral prostatectomy	24 (10.34)	29 (13.0)	38 (18.81)^a^	0.04
Transurethral removal of obstruction from ureter and renal pelvis	23 (9.91)	27 (12.11)	22 (10.89)	0.76
Radical prostatectomy	26 (11.21)	23 (10.31)	16 (7.92)	0.50
Complete nephrectomy	23 (9.91)	12 (5.38)	21 (10.4)	0.12
Other procedure	105 (45.25)	95 (42.6)	75 (37.13)	0.22

^a^ *P* < 0.05, compared with Period 1.

### Antimicrobial utilization

Pre-procedure antimicrobial data are shown in Table 2. In Period 1, cefuroxime was the most frequently prescribed pre-procedure agent (60.34%), with a median of 2 doses (IQR 1–3) and 1 day of therapy (IQR 1–2). Following intervention, fosfomycin became the predominant agent, prescribed in 53.36% and 84.16% of patients in Periods 2 and 3, respectively (*P* < 0.001). The median number of pre-procedure antimicrobial doses declined from 2 (IQR 1–3) in Period 1 to 1 (IQR 1–1) in Period 3 (*P* < 0.001). Although gentamicin was included in the institutional guideline, it was not prescribed in any patient during the study period. Similarly, while rectal swab–guided targeted prophylaxis was included as an option in the institutional guideline, no patients underwent rectal swab culture.

**Table 2.** Antimicrobials prescribed by study period

	Period 1 (n = 232)	Period 2 (n = 223)	Period 3 (n = 202)	*P*
Pre-procedure during admission				
Doses of antimicrobial (IQR)	2 (1–3)	1 (1–2)	1 (1–1)	<0.001
Days of therapy (IQR)	1 (1–2)	1 (1–1)	1 (1–1)	<0.001
Antimicrobial agents, n (%)				
Amoxicillin/clavulanate	3 (1.29)	2 (0.90)	4 (1.98)	0.63
Cefazolin	5 (2.16)	9 (4.04)	1 (0.50)	0.05
Ceftriaxone	32 (13.79)	26 (11.66)	13 (6.44)^a^	0.04
Cefuroxime	140 (60.34)	49 (21.97)^a^	1 (0.50)^a^	<0.001
Levofloxacin	17 (7.33)	3 (1.35)^a^	2 (0.99)^a^	<0.001
Fosfomycin	0	119 (53.36)^a^	170 (84.16)^a^	<0.001
Ertapenem	12 (5.17)	6(2.69)	21 (3.2)	0.08
Meropenem	11 (4.74)	4 (1.79)	3 (1.49)	0.07
Metronidazole^b^	29 (12.5)	5 (2.24)^a^	4 (1.98)^a^	<0.001
Others	18 (7.76)	7 (3.14)	4 (1.98)^a^	0.01
No antimicrobials	1 (0.43)	0	3 (1.49)	0.13
Post-procedure during admission				
Doses of antimicrobial (IQR)	6 (3–9)	3 (0–7)^a^	3 (0–6)^a^	<0.001
Days of therapy (IQR)	2 (2–4)	1 (0–3)^a^	1 (0–2)^a^	<0.001
Antimicrobial agents, n (%)				
Amoxicillin/clavulanate	4 (1.72)	5 (2.24)	2 (0.99)	0.60
Cefazolin	6 (2.59)	6 (2.69)	0	0.07
Ceftriaxone	29 (12.50)	25 (11.21)	13 (6.44)	0.10
Cefuroxime	130 (56.03)	50 (22.42)^a^	1 (0.50)^a^	<0.001
Levofloxacin	14 (6.03)	3 (1.35)^a^	1 (0.50)^a^	0.001
Fosfomycin	0	45 (20.18)^a^	95 (47.03)^a^	<0.001
Ertapenem	12 (5.17)	6 (2.69)	21 (3.2)	0.08
Meropenem	17 (7.33)	7 (3.14)	3 (1.49)^a^	0.01
Aminoglycoside	0	1 (0.45)	1 (0.5)	0.58
Metronidazole^b^	7 (3.02)	3 (1.35)	3 (1.49)	0.37
Others	10 (4.31)	9 (4.04)	7 (3.47)	0.90
No antimicrobials	11 (4.74)	70 (31.39)^a^	80 (39.60)^a^	<0.001
Discharge antimicrobials				
Doses of antimicrobial (IQR)	20 (8–20)	0 (0–20)^a^	0 (0–0)^a^	<0.001
Day of therapy (IQR)	5 (5–7)	0 (0–5)^a^	0 (0–0)^a^	<0.001
Antimicrobial agents, n (%)				
Amoxicillin/clavulanate	5 (2.16)	9 (4.04)	4 (1.98)	0.34
Cefixime	157 (67.67)	83 (37.22)^a^	22 (10.89)^a^	<0.001
Ciprofloxacin	4 (1.72)	2 (0.90)	1 (0.50)	0.44
Fosfomycin	2 (0.86)	0	1 (0.50)	0.39
Ertapenem	4 (1.72)	2 (0.90)	0	0.17
Levofloxacin	19 (8.19)	5 (2.24)^a^	4 (1.98)^a^	0.001
Others	8 (3.45)	7 (3.14)	3 (1.49)	0.41
No antimicrobials	36 (15.52)	118 (52.02)^a^	168 (83.17)^a^	<0.001
Total days of therapy (IQR)	10 (8–12)	5 (2–9)^a^	2 (1–4)^a^	<0.001
Single day of antimicrobial prophylaxis, n (%)	3 (1.25)	58 (25.11)^a^	73 (36.14)^a^	<0.001
Single dose of antimicrobial prophylaxis, n (%)	3 (1.29)	54 (24.22)^a^	68 (33.66)^a^	<0.001

^a^ *P* < 0.05, compared with Period 1.

^b^In combination with beta-lactams

Previous urine culture results were available for 46 of 232 patients (19.8%) in Period 1, 62 of 223 patients (27.8%) in Period 2 and 47 of 202 patients (23.3%) in Period 3. Among patients with a previous urine culture, bacterial growth was identified in 20 (43.5%), 26 (41.9%) and 22 (46.8%) patients during Periods 1, 2 and 3, respectively. The proportion of patients who received AP that was active against ceftriaxone-resistant organisms (carbapenems or fosfomycin) identified in the previous urine culture increased from 4 of 14 patients (28.6%) in Period 1 to 11 of 13 (84.6%) in Period 2 and 11 of 12 (91.7%) in Period 3 (*P* < 0.001).

Post-procedure antimicrobial use during admission decreased significantly. The median post-procedure DOT declined from 2 days (IQR 2–4) in Period 1 to 1 day (IQR 0–2) in Period 3 (*P* < 0.001). Among patients undergoing urological procedures, the proportion receiving no post-procedure antimicrobials increased from 4.74% in Period 1 to 39.6% in Period 3 (*P* < 0.001). The proportion of patients receiving no discharge antimicrobials also increased from 15.52% in Period 1 to 83.17% in Period 3 (*P* < 0.001). The total median DOT per admission decreased from 10 days (IQR 8–12) in Period 1 to 2 days (IQR 1–4) in Period 3 (*P* < 0.001). Single-dose AP increased from 1.29% in Period 1 to 24.22% in Period 2 and 33.66% in Period 3 (*P* < 0.001).

### Interrupted time series analysis

**Figure 2.**
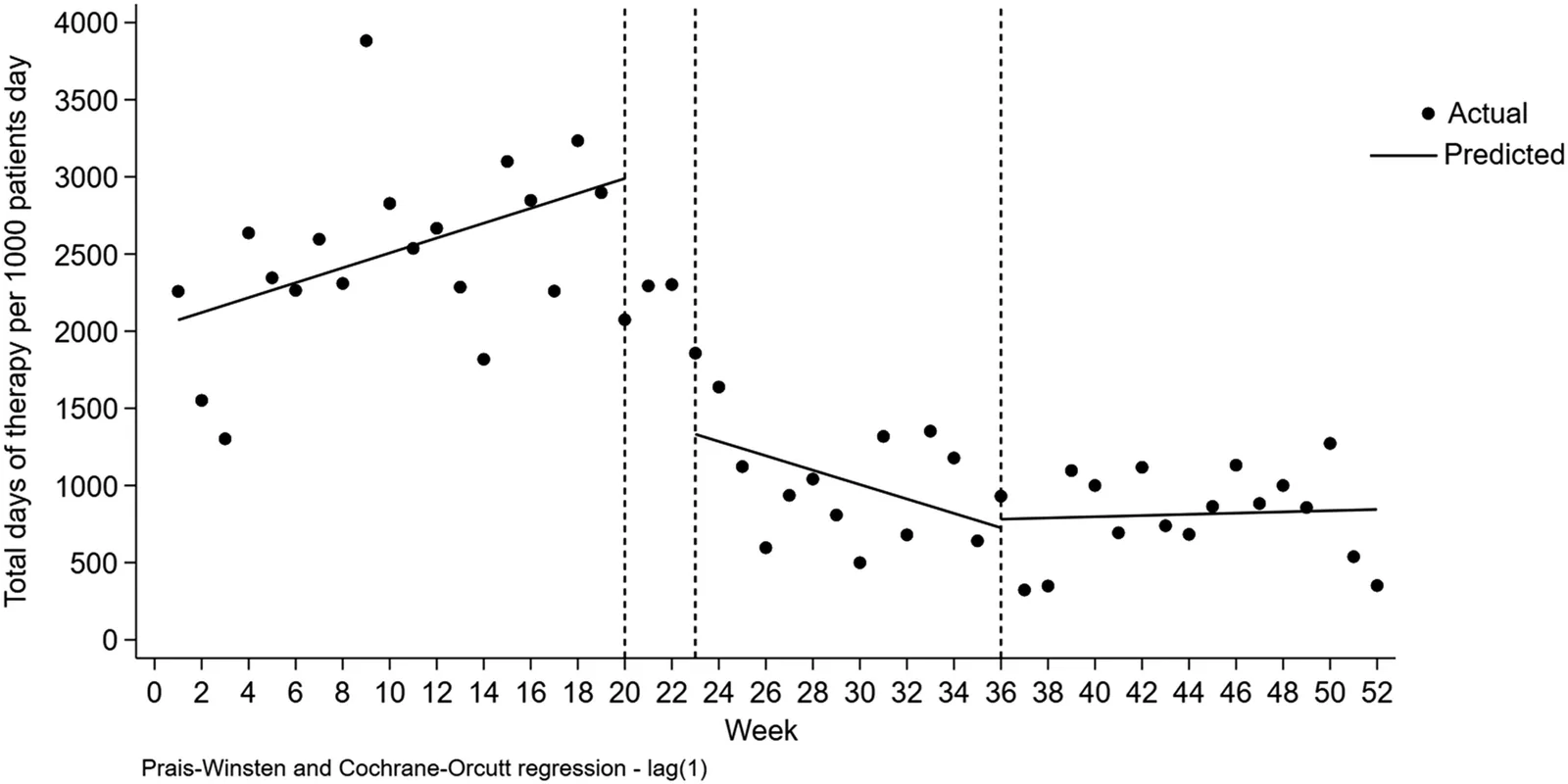
Total days of therapy per 1000 patient-days per week of antimicrobial prophylaxis.

**Figure 3.**
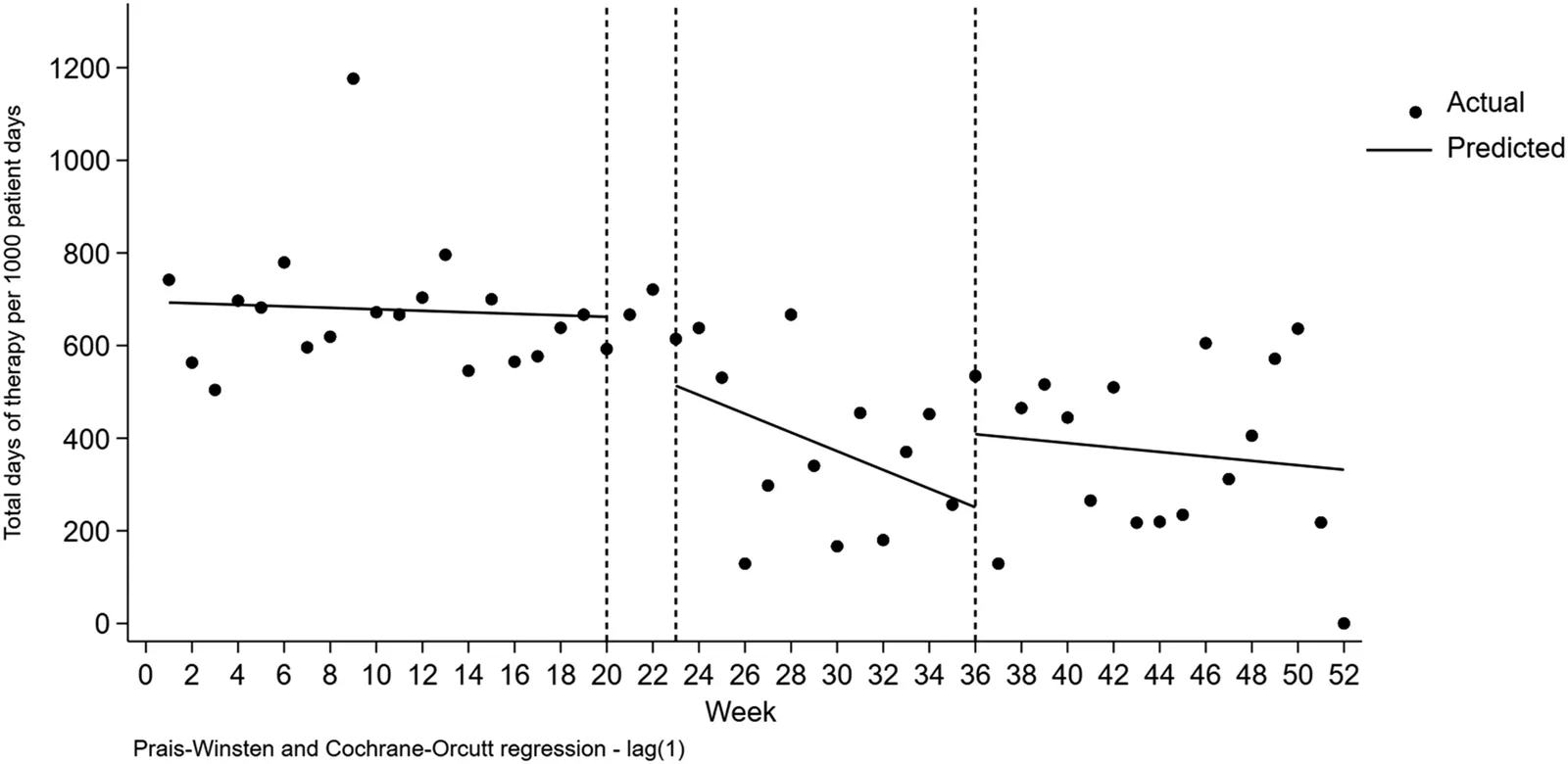
Days of therapy per 1000 patient-days per week of antimicrobial administration post-urological procedure during admission.

ITS analysis on 52 weekly observations identified a significant immediate level reduction in total DOT per 1000 patient-days comparing Period 2 to Period 1 (level change: −3156.8; 95% CI −4604.7 to −1708.9; *P* < 0.001), with no significant change in slope (Table 3, Figure 2). A significant level reduction was similarly observed in post-procedure DOT per 1000 patient-days (level change: −974.8; 95% CI −1536.6 to −412.9; *P* = 0.001) (Table 3, Figure 3). Sustained significant reductions were observed in Period 3 versus Period 1 for both total DOT (level change: −2016.0; 95% CI −2792.2 to −1241.6; *P* < 0.001) and post-procedure DOT (level change: −535.2; 95% CI −817.9 to −252.4; *P* < 0.001). No significant changes in slope were observed across any comparison.

**Table 3.** Change in post-urological procedure and total days of therapy per 1000 days per week of antimicrobial from interrupted time series analysis, with segmented regression analysis during the entire duration of the study

Period comparison	Change in y-intercept		Change in slope	
	Mean value (95% CI)	*P*	Mean value (95% CI)	*P*
Period 2 versus Period 1				
Post-urological procedure DOT per 1000 patient-days per week	−974.75 (−1536.56 to −412.94)	0.001	−85.27 (−325.24 to 154.71)	0.48
Total DOT per 1000 patient-days per week	−3156.79 (−4604.66 to −1708.92)	<0.001	−195.97 (−809.54 to 417.59)	0.53
Period 3 versus Period 2				
Post-urological procedure DOT per 1000 patient-days per week	439.58 (−126.43 to 1005.60)	0.13	102.3 (−140.86 to 345.46)	0.40
Total DOT per 1000 patient-days per week	1139 (−327.71 to 2607.45)	0.13	198.28 (−427.85 to 824.41)	0.53
Period 3 versus Period 1				
Post-urological procedure DOT per 1000 patient-days per week	−535.17 (−817.92 to −252.43)	<0.001	17.03 (−14.81 to 48.88)	0.29
Total DOT per 1000 patient-days per week	−2016 (−2792.23 to −1241.62)	<0.001	2.31 (−85.99 to 90.60)	0.96

Abbreviations: DDD, defined daily doses; DOT, days of therapy.

### Postoperative infectious complications following urological procedures

The rates of UTI and SSI within 30 days after urological procedures remained low and stable across all three study periods. UTI occurred in two patients (0.9%) during Period 1, decreasing to one patient (0.5%) in Periods 2 and 3. No SSIs were recorded in Period 1. However, one case (0.4%) was identified during Period 2 and one case (0.5%) in Period 3. Microbiologic data showed no consistent resistance pattern: one UTI isolate was ceftriaxone-susceptible but fluoroquinolone-resistant, one showed mixed growth in the setting of staghorn calculus and one was pan-susceptible; among SSI cases, one was MSSA, and one had no growth.

## Discussion

This study demonstrates that embedding a locally adapted AP guideline into standardized pre-procedure order sets, supported by multifaceted ASP interventions, was associated with a significant reduction in antimicrobial exposure among patients undergoing urological procedures. An abrupt level-change reduction in total DOT per 1000 patient-days without a significant change in slope indicated a pattern characteristic of policy-driven intervention that alters prescribing defaults through standardized workflows, rather than gradual behaviour change.^18^ The reduction was sustained in Period 3 without ongoing active intervention, suggesting that the order sets helped establish durable prescribing norms once a culture shift was initiated.

The baseline prescribing pattern in Period 1 exemplified the challenge of inappropriate AP in LMIC settings. Cefuroxime was prescribed in 60.34% of cases despite susceptibility rates lower than 60% against local *E. coli* isolates, and single-dose regimens were used in fewer than 2% of patients. Adherence to AP guidelines has been reported below 10% in LMICs compared with 49%–64% in high-income countries.^2,3,4,19^ Furthermore, LMICs are disproportionately affected by inappropriate agent selection in addition to prolonged duration.^2,20^ Our interventions target both the duration of therapy and the selection of appropriate agents guided by local resistance patterns.

The post-intervention shift to fosfomycin reflects deliberate alignment with the local antibiogram, given its high susceptibility among local *E. coli* isolates and its limited use in therapeutic settings. The rationale for selecting fosfomycin as a primary prophylactic agent in urological procedures is supported by its broad Gram-negative activity, including against ESBL-producing organisms,^15^ and evidence supporting its use as antimicrobial prophylaxis in urology in settings with high antimicrobial resistance.^16,17^ Gentamicin and amoxicillin/clavulanate were included as alternatives for selected procedures and risk profiles. Although the predominance of fosfomycin and gentamicin may limit generalizability, the core principle of agent selection accounting for local pathogen prevalence, susceptibility profiles and therapeutically spared agents is broadly applicable to any high-resistance setting and aligns with current consensus recommendations.^6,7^

The designed implementation in this study addressed barriers at multiple levels. At the team level, engaging the urology division from the initial drafting phase fostered clinical ownership and built collaborative trust between surgical and infectious diseases teams. This step intended to mitigate the perception that ASP constrains provider autonomy, which could potentially create negative attitudes and weaken collaborative rapport.^21,22^ At the clinical decision-making level, the one-page order sets eliminated the need for individual prescribers to recall or locate guideline recommendations at the time of prescribing, reducing cognitive burden and logistical friction of locating guidelines in real time. This is consistent with evidence suggesting that structural simplification of prescribing processes is more effective than passive guideline dissemination.^23,24^ Active education, a messaging application group that enabled regular reminding, rapid feedback and consultation were layered in to maintain prescriber engagement through the rollout period. This approach aligns with systematic reviews of AMS interventions on surgical AP in LMICs, which found that multifaceted approaches appear to be effective in promoting adherence.^25,26^

Postoperative UTI and SSI rates remained below 1% across all study periods, which may be viewed as safety signals. Because infection events were few, meaningful statistical comparisons are not possible.

Patient age and the number of transurethral prostatectomy procedures differed significantly between the study periods. There were no changes in the admission system or surgical scheduling practices during the study period. However, the reasons for these differences were not investigated as part of this study and therefore remain unknown.

This study has several limitations. First, the study design, while appropriate for evaluating interventions, is subject to residual confounding. Second, although the intervention targeted prescribing behaviour rather than specific procedures, heterogeneity in surgical procedures may have influenced antimicrobial utilization and the adequacy of coverage. These order sets prioritized Gram-negative coverage, as institutional SSI surveillance data demonstrated a predominance of Gram-negative organisms. However, gentamicin may not provide optimal coverage for procedures at risk of skin flora contamination, such as those involving skin incision or prosthetic implantation. This underscores the importance of procedure-specific prophylaxis and the need to refine local guidelines to better align antimicrobial selection with procedural risk profiles. Third, the post-intervention observation period was limited to 4 months. The durability of prescribing change beyond this window is unknown. Future studies should evaluate procedure-specific optimization, long-term sustainability and postoperative outcomes.

In conclusion, embedding locally tailored antimicrobial prophylaxis guidelines into standardized pre-procedure workflows can substantially reduce unnecessary antimicrobial exposure in urological surgery. This feasible stewardship approach could be adopted, particularly in high-resistance, resource-limited settings.

## Supplementary Material

dlag202_Supplementary_Data

## Supplementary data

Figures S1 and S2 are available as Supplementary data at *JAC-AMR* Online.
