# Supplementary material for: Embedding locally tailored antimicrobial prophylaxis into standardized order sets for urological procedures reduces antimicrobial use
Source: JAC Antimicrob Resist. 2026 Sep 3;8(5):dlag202. doi: 10.1093/jacamr/dlag202 (PMC13539358; doi:10.1093/jacamr/dlag202)
Supplement: Supplementary file 1 [file dlag202_supplementary_data.docx]

Supplementary data

**Figure S1.** The institutional antibiogram from clinical isolates (January - December 2023)

| Organisms | Number of isolates | Amikacin | Gentamicin | Amoxycillin/clavulanic acid | Piperacillin/tazobactam | Cefoxitin | Cefuroxime | Ceftriaxone | Cefepime | Ertapenem | Meropenem | Ciprofloxacin | Levofloxacin | Trimethoprim/sulfamethoxazole | Fosfomycin |
| --- | --- | --- | --- | --- | --- | --- | --- | --- | --- | --- | --- | --- | --- | --- | --- |
| *E. coli* | 3281 | 99 | 71 | 82 | 94 | 86 | 59 | 64 | 87 | 98 | 99 | 38 | 43 | 51 | 97 (87) |
| *K. pneumoniae* | 1509 | 95 | 86 | 71 | 79 | 75 | 59 | 66 | 97 | 88 | 92 | 50 | 58 | 61 | 86 (28) |
| *P. mirabilis* | 485 | 100 | 69 | 93 | 99 | 96 | 84 | 87 | 98 | 100 | 100 | 57 | 57 | 52 | 91 (11) |
| *E. cloacae* | 271 | 100 | 89 | R | 80 | R | 47 | 72 | 91 | 94 | 97 | 72 | 80 | 77 | 80  (5) |
| *C. koseri* | 147 | 100 | 98 | 97 | 99 | 92 | 85 | 98 | 100 | 100 | 100 | 97 | 97 | 99 | 100  (6) |

Note. Fosfomycin disc susceptibility testing was performed on 151 urine samples collected

during October–November 2023, and the number of isolates for each organism is presented in parentheses in the table.

**Figure S2.** One-page order sets

Name........................................................................

HN…………………………………………………………………..

Age.............................. Page................................

| **Department** | **Division** | | **Ward** |
| --- | --- | --- | --- |
| **Attending Staff** | | **Resident** | |

DOCTOR’S ORDER SHEET

Pre-operative Physician order sheet (Urological procedures)

Diagnosis________________________________________________________Admit date__________________

Procedure_______________________________________________________Operative date_______________

| **Date**  **Hour** | **Order for one day** | **Date**  **Hour** | **Orders for Continuation/ Surgical related order** |
| --- | --- | --- | --- |
|  | **Pre-operative order for urological procedure**  Lab  🞎 CBC 🞎 BUN, Cr, Electrolytes  🞎 Anti-HIV 🞎 PT, PTT, INR (optional)  🞎 Fasting plasma glucose 🞎 HbA1C  🞎 Urinalysis 🞎 EKG 12 leads  🞎 Chest X-ray  🞎 Others……………………………………………………….  Blood components  🞎______________________________________  🞎 NPO after 🞎 midnight 🞎……………………………  **IV fluid** 🞎__________________rate………….mL/h  start IV fluid 🞎 After NPO 🞎 on the morning of  procedure  🞎 POCT glucose q……….h after NPO  Keep ……………………………………………………………  Other………………………………………………………………………...  …………………………………………………………………………………  ………………………………………………………………………………..  Sign……………………………………..Code…………………. |  | Position………………………………………………………………………………….  Mark site 🞎 No 🞎 Yes………………………………….........................  Prep skin 🞎 No 🞎 Yes………………………………...........................  Drug to be discontinued……………………………………………………...  **Antimicrobial prophylaxis before urology procedures**  🞎 **Endoscopic procedure and any biopsy *NOT* involving the**  **intestine**  🞎 **Percutaneous nephrolithotomy (PCNL)**  🞎 **Robotic assist (radical prostatectomy, cystectomy)**  ⭘Fosfomycin 4 g IV 1 dose OR  ⭘Gentamicin (in eGFR > 20 mL/min) 5 mg/kg  =………………….mg IV 1 dose  🞎 **Transrectal prostate biopsy**  ⭘Base on rectal swab or stool swab  ………………………………………………………………………...  ⭘No rectal swab  ⭘ Fosfomycin 4 g IV q 8h for 24h OR  ⭘Gentamicin (in eGFR > 20 mL/min) 5 mg/kg  =……………….mg IV 1 dose  🞎 **Urology procedures involving the intestine**  ⭘ Amoxicillin/clavulanate 1.2 g IV 1 dose OR  ⭘ Gentamicin (in eGFR > 20 mL/min) 5 mg/kg  =……………….mg IV + Metronidazole 500 mg IV 1 dose  🞎 **Transrectal prostate biopsy in *outpatient setting***  ⭘ Amoxicillin/clavulanate (1g) 1 tab PO 1 dose 2-4h before  procedure, then q 12**h for** 48h (total 4 doses**)**  ⭘ Fosfomycin trometamol (3g) 1 sachet + water 150 mL PO  1 dose 2-4h before and 24h after procedure (total 2 doses)  🞎 Other……………..............................dose…………………….………………….  duration…………………………., reason…………………………………………………  **S**ign…………………………………………………… Code……………………... |
|  | **Pre-Anesthetic Order**  …………………………………………………………………………………  …………………………………………………………………………………  …………………………………………………………………………………  ………………………………………………………………………………...  …………………………………………………………………………………  …………………………………………………………………………………  Sign………………………………………………...Code……………….. |  |  |
